# Childhood vaccination among Polish immigrants in Norway: a qualitative study

**DOI:** 10.1186/s12889-024-19426-5

**Published:** 2024-07-24

**Authors:** Rebecca Nybru Gleditsch, Kamila Hynek, Bo T. Hansen, Trine Skogset Ofitserova, Brita Askeland Winje, Thea Steen Skogheim

**Affiliations:** 1 Department of Infection Control and Vaccine, Norwegian Institute of Public Health, PO Box 222, Skøyen, Oslo NO-0213, Norway; 2 Fafo Institute for Labour and Social Research, PO Box 2947, Tøyen, Oslo NO-0608, Norway; 3 Verian, Lakkegata 23, Oslo, NO-0130 Norway; 4 Oslo Meteropolitan University, St. Olavs plass, PO Box 4, Oslo, NO-0130 Norway; 5 Cluster for Health Services Research, Norwegian Institute of Public Health, PO Box 222, Skøyen, Oslo, N-0213 Norway

**Keywords:** Childhood vaccination, Immunization programs, Immigrants, Norway, Poland, Qualitative research, Interviews, Thematic analysis, Vaccine hesitancy, vaccine readiness

## Abstract

**Background:**

Confidence in childhood vaccination is high in Norway and the Norwegian Childhood Immunization Programme (NCIP) achieves high overall coverage rates. However, lower coverage has been observed for some immigrant groups, including Polish immigrants who represent the largest immigrant group in Norway. Anti-vaccine sentiments and increased refusal of mandatory childhood vaccination has been on the rise in Poland, but it is unknown whether such attitudes also apply to Polish immigrants in Norway, as they experience a different vaccination policy and perhaps also different attitudes to vaccines. This qualitative study aims to explore attitudes towards childhood vaccination in Norway among Polish immigrants.

**Methods:**

We interviewed 15 Polish parents living in Norway in 2022. We recruited the participants by purposive sampling and analyzed the interviews by reflexive thematic analysis.

**Results:**

Three themes were identified: views of childhood vaccination, vaccine hesitancy, and differences in childhood vaccination between Poland and Norway. Overall, the participants favored childhood vaccination and viewed most of the vaccines included in the NCIP as safe and reliable. Human papilloma virus, meningococcal and pneumococcal vaccines were declined by some of the parents. Comparisons of childhood vaccination in Poland and Norway was evident in many of the interviews, especially among parents whose children had received vaccines in both countries. The participants were well acquainted with the NCIP, favored voluntary childhood vaccination, and the majority expressed a high level of trust in Norwegian health authorities.

**Conclusions:**

Polish immigrants to Norway generally expressed positive views about childhood vaccination. Non-vaccination was related to lack of knowledge and/or unfamiliarity with certain vaccines and not with anti-vaccine sentiments or conspiracy theories. The study highlights how parents’ knowledge, in combination with norms and trends from both birth country and country of residence, influence parents’ decision making about vaccination.

## Background

The success of an immunization program depends on high coverage rates in the target population. However, high overall coverage may mask suboptimal uptake in small communities and minority groups, which can lead to disease outbreaks among unvaccinated individuals and puts children in these groups at risk of vaccine-preventable diseases. The Norwegian Childhood Immunization Program (NCIP) has very high overall coverage rates for the vaccines included in the program and low incidences of the targeted diseases [1], but relatively low coverage has been observed for some vaccines in some immigrant groups [2, 3]. Moreover, measles outbreaks have occurred in unvaccinated communities [4, 5], and a lower level of timely childhood vaccination has been documented among immigrants [6]. Vaccination in the NCIP is free of charge and distributed at child health centers and school health services. The program is recommended to all parents and equity in uptake is a priority. However, parents can choose whether their children should receive vaccines, and vaccine refusal is not sanctioned. Thus, equity in uptake relies on similar levels of trust in vaccination and accessibility of vaccines across sociodemographic backgrounds.

The World Health Organization (WHO) lists vaccine hesitancy as one of ten threats to global health [7]. WHO defines vaccine hesitancy as the “*delay in acceptance or refusal of vaccines despite availability of vaccine services. Vaccine hesitancy is complex and context specific*,* varying across time*,* place*,* and vaccines. It is influenced by factors such as complacency*,* convenience*,* and confidence*” [8]. Higher vaccine hesitancy may partly explain the suboptimal vaccine uptake observed in some immigrant groups [9, 10]. In addition, systemic, organizational, and cultural factors such as language may act as barriers to immigrants´ vaccine uptake [11, 12].

Poles represent the largest immigrant group in Norway. As of 2023, nearly 108 000 persons with Polish background had moved to Norway, which has a total population of 5.5 million [13]. The majority of Polish immigrants in Norway are males that arrived after Poland joined the European Union (EU) in 2004, and many are labor migrants filling positions in construction, manufacturing, and services [14]. The recent pandemic revealed that Poles were among the immigrant groups with the lowest COVID-19 vaccine coverage in Norway [15]. The relatively low COVID-19 vaccine coverage among Poles in Norway may to some extent reflect the situation in Poland, where anti-vaccine sentiments and increased refusal of mandatory childhood vaccination recently have been on the rise [16], and the coverage of COVID-19 vaccines ranges low among European countries [17]. A recent survey showed that Polish immigrants in Norway held less positive attitudes to COVID-19 vaccinations than Norwegians, while they did not differ in this respect from Poles living in Poland [18]. Moreover, Polish immigrants had lower trust in the Norwegian healthcare system compared to Norwegians [18] and compared to other immigrant groups in Norway [19]. Conspiracy beliefs about vaccines also appear to be relatively common among Polish immigrants in Norway [20]. In addition, it has been shown that language may act as a barrier and that there may be a need for more translated COVID-19 information to immigrants in Norway [21].

The legal terms of childhood vaccination differ between Poland and Norway. Vaccination in the NCIP is voluntary, while childhood vaccination is obligatory for most vaccines in the Polish Childhood Immunization Program (PCIP) (Tables 1 and 2), and parents who do not comply to vaccination in Poland may be fined [22]. We do not know whether the hesitancy towards COVID-19 vaccination that is evident among Polish immigrants in Norway also may be relevant for Polish immigrants in the context of childhood vaccination in the NCIP. The vaccines offered through the NCIP differ from the COVID-19 vaccines in several ways that may impact on trust in the vaccines and in the system that delivers them. For instance, the NCIP vaccines are not based on novel mRNA technology, and their effectiveness and safety have been tested over a longer period. They are offered through child health centers and school health services, as part of the children’s regular health and development consultations. Confidence in childhood vaccination is high in Norway [23] and the overall coverage remained high even through the pandemic [1]. In contrast, confidence in childhood vaccination seems far lower in Poland [22, 24]. It is unknown whether such attitudes to childhood vaccination also apply to Polish immigrants in Norway, who experience a different vaccination policy and perhaps also a different attitude to vaccines among the majority population.

**Table 1:** Norwegian Childhood Immunization Program, 2023* [25]

	Weeks	Months					Years				
	6	3	5	6	12	15	7	11	12	15	18
BCG (Tuberculosis)	D1^a^										
Rotavirus	D1	D2									
Diphtheria, Tetanus, Pertussis, Hepatitis B, Poliomyelitis, Haemophilus influenza type B		D1	D2		D3						
Pneumococcal		D1	D2		D3						
MMR (Measles, Mumps and Rubella)						D1		D2			
Diphtheria, Tetanus, Pertussis, Poliomyelitis							D1			D2	
HPV (Human Papillomavirus)									D1		
Meningococcal											D1^b^
Influenza		Yearly from 6 months ^a^									

^*^ Timing of doses (D1 = dose 1, etc.) of vaccines associated with the NCIP. ^a^ Defined risk groups. ^b^ Not part of the NCIP but recommended for 16–19-year-olds

**Table 2:** Polish childhood immunization program, 2023* [26]

		Months						Years			
	Birth	2	3–4	5–6	7	13–15	16–18	6	12–13	14	19
Tuberculosis^a^	D1										
Hepatitis B^a^	D1	D2			D3						
Rotavirus^a^		D1	D2	D3							
Diphtheria, Tetanus, Pertussis^a^		D1	D2	D3			D4	D5		D6	
Tetanus, Diphtheria											D1
Haemophilus influenza type B^a^		D1	D2	D3			D4				
Poliomyelitis^a^			D1	D2			D3	D4			
Pneumococcal^a^		D1	D2			D3					
MMR (Measles, Mumps, Rubella) ^a^						D1		D2			
HPV (Human Papillomavirus)									D1		
Influenza				Yearly from 6 months							
Meningococcal C		D1 at 2–6 months			D2 at 7 months – 19 years						
Varicella						9 months – 12 Years^a^					
Hep A						9 months – 12 Years^a^					
TBE						9 months – 12 Years^a^					

* Timing of doses (D1 = dose 1, etc.) of vaccines associated with the PCIP. ^a^ Mandatory. ^b^ Defined risk groups

In the present study, we performed qualitative interviews with Polish immigrants in Norway with the objective to address their attitudes towards childhood vaccination in Norway.

## Methods

### Study design and procedures

This study was part of a larger project exploring attitudes towards vaccination among immigrants in Norway. Poland was among the country backgrounds chosen because previous studies have found lower coverage for both adult vaccines such as COVID-19 vaccine [15], as well as childhood vaccines such as HPV [3], pertussis and MMR [27]. We performed individual semi-structured interviews with Polish immigrants in Norway. The interview guide was developed in collaboration with researchers at the Norwegian Institute of Public Health (NIPH). In order to explore the participants’ views and attitudes towards vaccination more broadly, specific themes emerging from past quantitative studies on vaccination in Norway [1–3, 6, 15, 18, 21, 27] were used to structure the interview guide (topics used to structure the guide were: knowledge about vaccines, trust, childhood vaccination, and COVID-19), while also leaving room for flexibility and adaptability during the interviews to ensure that each participant could share their experiences and views. The interview guides were piloted with two individuals with Polish background and revised. Purposive sampling was used to recruit participants, utilizing informal networks through Polish organizations, Polish schools, and Polish-speaking churches. Some of these organizations forwarded information about the study through email and private Facebook groups, while others distributed posters or flyers. Adults 18 or older, living in Norway, and self-identifying as being from Poland were eligible for participation.

### Data collection

A total of 15 semi-structured interviews were conducted by KH. Recruitment was stopped after 15 participants as no new themes or information emerged from the interviews. All interviews were conducted between September and November 2022. The participants could opt for interview in Polish, English or Norwegian, however, all interviews were done in Polish. The interviews took place face-to-face (*N* = 14) at venues chosen by the participants, such as cafés, libraries, or meeting rooms at a local school. One interview was done digitally by Microsoft Teams at the request of the participant. All participants signed consent forms prior to the interview. Each individual interview lasted between 45 and 60 min. The interviews were audio recorded and later transcribed and translated verbatim by the second author (KH). Three of the transcripts were quality assessed by a non-member of the research team. Each participant received a $30 gift card to compensate for any expenses or inconvenience associated with the interview participation.

### Data analysis

The data was inductively analyzed using a reflexive thematic analysis (TA) to develop and interpret patterns of meaning across the interviews [28]. We followed the six phases of reflexive TA as outlined by Braun & Clarke [28]. Two researchers that had not taken part in the interviews (RG and TSS) analyzed the data. The first step of the data analysis consisted of becoming familiar with the data by reading the interview transcripts and making note of preliminary ideas and thoughts. Each transcript was coded by RG and TSS independently to increase reliability and enhance understanding, interpretation, and reflexivity in the data analysis process [28]. The codes identified by each researcher were compared before they were reanalyzed by the research team to generate, refine, and define themes across the interviews. The themes were reviewed, developed, and discussed with the interviewer (KH) to compare with her experiences with each interview. Furthermore, the themes were refined, defined, and named, this occurred both prior to and during the writing phase of the present article. All names have been replaced with pseudonyms and any identifying details have been removed or changed. The qualitative analysis software NVivo was utilized for the entire data analysis process.

## Results

A total of 15 participants were interviewed in this study (Table 3), of which five were men and 10 were women. Their age ranged from early 30s to late 60s. Most (*N* = 12) had completed higher education (college or university). The majority of the participants (*n* = 11) had lived more than 10 years in Norway. All participants had at least one child and all, but two, were married.

**Table 3:** Characteristics of participants (*n* = 15)

Pseudonym	Gender	Age	Country of birth	Education level	Years in Norway	Marital status*	Children	Age children
Aleksandra	Female	40–49	Poland	Higher	20+	Married	1	6–20
Andrzej	Male	30–39	Poland	Higher	5–9	Married	2	6–20
Elzbieta	Female	30–39	Poland	Secondary	10–19	Married	2	6–20
Ewa	Female	40–49	Poland	Higher	10–19	Married	3	6–20
Jan	Male	50–59	Poland	Higher	0–4	Married	2	6–20, adult
Katarzyna	Female	30–39	Poland	Higher	0–4	Married	2	0–5
Krystyna	Female	50–59	Poland	Higher	10–19	Single	1	Adult
Magdalena	Female	30–39	Poland	Higher	10–19	Married	3	0–5
Małgorzata	Female	40–49	Poland	Higher	10–19	Married	3	6–20, adult
Monika	Female	40–49	Poland	Higher	10–19	Married	2	0–5
Pawel	Male	30–39	Poland	Secondary	0–4	Married	2	0–5, 6–20
Piotr	Male	40–49	Poland	Secondary	10–19	Single	3	6–20, adult
Teresa	Female	40–49	Poland	Higher	10–19	Married	1	0–5
Tomasz	Male	60–69	Poland	Higher	20+	Married	2	Adult
Zofia	Female	40–49	Poland	Higher	20+	Married	2	0–5

*Married or cohabitant

Three main themes were identified in the data, (i) views of childhood vaccination, (ii) vaccine hesitancy, and (iii) differences in childhood vaccination between Poland and Norway.

### Theme 1: “As I see it, these vaccines are not offered without reason”

The majority of the participants shared favorable views of routine childhood vaccines and described being vaccinated in Poland as a child. When asked about the decision-making process leading up to vaccination, many seemed to view vaccinating their children as a given, stating that protection against diseases was the main reason for their choice:

*I want my children to be vaccinated. I myself was vaccinated as a child and thanks to this there are many diseases I have not had to undergo. So I wanted my children to be vaccinated too.* (Katarzyna)

Regardless of how long the participants had lived in Norway, their descriptions of the NCIP indicated that most were well-acquainted with the program. Their children had either partly or completely taken part in the program and among those whose children had received vaccination in both countries, the transition from the Polish Immunization program to the Norwegian seemed to have been unproblematic:

*Before we moved*,* my oldest child began the vaccination program in Poland*,* and we followed all recommendations. Later*,* when we moved here [to Norway]*,* the children followed the Norwegian program. And of course*,* my second child was born here*,* and it was a little different as we immediately followed the Norwegian [immunization] program.* (Ewa)

Several emphasized that having their children vaccinated was facilitated by the organization of vaccination through their local health care centers during their children’s regular child health program provided by public health nurses. Trusting the vaccines and believing that both the vaccines and recommendations were safe were important factors in their decision-making process:

*I am not worried about vaccines that are taken regularly by my children. Because all children take these. I also know that if my child is sick [at the time of vaccination] they will not be vaccinated because that is how the recommendations are. When I think about the children’s [vaccines] I am much more… I think I feel safe about the vaccines that are given.* (Katarzyna)

The majority of the participants emphasized having a high level of trust in Norwegian health authorities. In addition to trusting official vaccination recommendations, most expressed that they trusted the vaccines and described vaccination as something their children received when they attended the regular consultations.

*As I see it*,* these vaccines are not offered without reason*,* therefore I have never had any objections*,* anxiety or need for additional information.* (Elzbieta).

*I don’t remember if it [vaccination] was something we discussed at home. We simply accepted what was recommended by the Norwegian health authorities without any major discussions.* (Tomasz)

Overall, the participants expressed high levels of trust in the vaccines offered to their children and described having few questions about the vaccines or potential side effects.

### Theme 2: “I don’t know anyone from Poland who has taken it”

While most of the participants stated that making a decision regarding childhood vaccination on behalf of their children was easy, some expressed hesitancy towards specific vaccines. Several of the participants expressed some degree of vaccine hesitancy, however, this was always related to a specific vaccine and not the NCIP or childhood vaccination in general. None of the participants had refused childhood vaccinations altogether, but a few of the participants expressed hesitancy related to the HPV, meningococcal and/or pneumococcal vaccines. For some, the hesitancy was due to unfamiliarity with the specific vaccine:

*For example*,* the HPV vaccine*,* [the child] didn’t take it*,* I don’t know anyone from Poland who has taken it. Because I have asked*,* we have acquaintances… while our Norwegian acquaintances*,* they all said that you had to because such and such. So*,* it’s basically two different worlds.* (Ewa).

Others expressed having doubts when their child was offered a vaccine that recently had been implemented in the NCIP and described uncertainty related to possible side effects of the vaccine:

*I know that when my child was little*,* there were vaccines that were new. I don’t know if it was pneumococcal or something like that*,* I don’t remember*,* but it was new to the program. I was a little doubtful because my child could get this vaccine*,* but didn’t have to*,* and there was still little data on this vaccine. There was no information on long-term effects [or] any side effects in people who took this vaccine.* (Aleksandra)

Some parents described being hesitant to vaccinate their children with specific vaccines when they viewed the likelihood of their child getting sick as minimal and the vaccine not being necessary:

*It [meningitis] is not familiar to me and you rarely hear about it. I know that there is a different culture here […] but it is also about trusting the child and how you raise them. For example*,* I know that my child will not be partying and share bottles with other people.* (Ewa)

While others expressed not being hesitant themselves, but hearing concerns from acquaintances, such as this mother who encountered a friend from Poland after having her own child vaccinated with the HPV-vaccine:

*But then I met a Polish woman who said ‘Oh my God*,* what have you done? These vaccines destroy the organism*,* she might have difficulties getting pregnant.’ […] I’m immediately [thinking] like ‘Oh*,* have I hurt my child?’ Because it’s a dilemma*,* but you must use common sense*,* in my opinion. You have to find a middle ground between what you hear from others and the knowledge you possess yourself.* (Malgorzata)

One participant also described how searching for information on the Internet contributed to her hesitancy, explaining that she felt like she read too much:

*I read too much on the Internet. “Doctor Google” has a very large library and lots of sources. When you start reading your [thinking] starts to get narrower.* (Ewa)

Two of the participants talked about their experiences when vaccinating their children in Poland before moving to Norway and encountering discussions related to “clean vaccines.” Although they were not able to fully explain what was meant by “clean” vaccines, this seemed to have made them uncertain about the specific vaccine and believing that the “unclean” version of vaccine was unsafe and would cause more side effects. One of the mothers we talked to, explained that her child had received the first dose and had been crying and having what she described as muscle contractions later the same day. When she contacted her doctor, she was told that this was common «[…] because it was not clean, but they [the doctor’s office] would make a note and ensure that the next dose given to the child would be a fully cleaned vaccine.” Although this experience did not seem to make her hesitant regarding other vaccines in neither the Polish nor Norwegian immunization program, both women emphasized wanting to ensure that future doses were “clean.”

### Theme 3: “Here in Norway they don’t force us to vaccinate”

Many of the individuals we talked to expressed feeling less pressure to be vaccinated in Norway compared to Poland. While all childhood vaccines in Norway are voluntary and free of charge, 11 of the 16 recommended childhood vaccines in Poland are mandatory by Polish law (Table 1). There was variation in the participants’ ability to compare the childhood vaccination programs in Norway and Poland. Some had moved to Norway with their children and had thus experienced both programs, while others had children who were born in Norway and had only experience with the NCIP. Having experience with both programs made some of the participants question the differences in recommended vaccines, exemplified by Teresa’s description:

*We asked [the public health nurse] about vaccines that are part of the Polish program but not here. For example*,* the vaccine against tuberculosis*,* which has always been part of the Polish child vaccination program. I don’t know if it’s still there*,* but it probably is. So*,* we asked if they should get this vaccine in addition. We have even talked about the children’s covid vaccine. But here in Norway*,* if I remember correctly*,* there was no program for vaccinating children*,* and the [covid] vaccine was only recommended for children with underlying diseases.* (Teresa)

Others who had limited experience with childhood vaccination in Poland were less focused on specific vaccines within the programs and viewed the key difference between the Polish and Norwegian childhood immunization program to be the differences in voluntary versus mandatory vaccination. Although none of the participants had refused taking part in the childhood vaccination program in any of the countries, several emphasized feeling less pressured to vaccinate when the vaccines are offered on a voluntary basis, as illustrated by Katarzyna:

*When it comes to [vaccine] recommendations*,* I think they are the same. It is always emphasized that it is better for the child to get vaccinated*,* for the benefit of their health and those around them. But I think there is more pressure from the pediatrician in Poland*,* like ‘just do it.’ While here in Norway*,* if I had said that I didn’t want my child to be vaccinated*,* I think the nurse would just have made a note that the parent does not give consent. It could be that the other parent would have to give the same statement about consent*,* but I don’t think anyone would punish me for that in any way*,* would not look suspiciously at me*,* would not force me. [They would] perhaps explain the benefits [of vaccination]*,* but it is very different when it comes to pressure to vaccinate in Poland and here in Norway.* (Katarzyna)

Not all the participants were familiar with the mandatory vaccination policies in Poland, but most were in agreement about preferring voluntary vaccination. However, a few of the individuals we talked to expressed favoring mandatory childhood vaccination, such as Magdalena:

*I didn’t know that it was mandatory in Poland*,* but I think that’s good*,* and I think that it should be mandatory for all children between 0 and 18 years in Norway too. Because certain things should not be decided by us parents*,* but by people who have knowledge about it. And of course*,* it has an impact [on vaccination] that not everyone has knowledge about this topic.* (Magdalena)

At the same time, not all had experienced the NCIP as completely voluntary, and this mother explained that the way the vaccines had been presented had made her feel like she had a duty to vaccinate her child:

*I don’t feel that [the NCIP is voluntary]*,* because the first vaccines that my younger child received*,* who was born in Norway*,* they were part of the childhood vaccination program. My child got them regularly and I received information about [them] and was told that I should contact them [health care center] if needed. I did not feel like it was voluntary. In a way I felt that even if it is in fact voluntary*,* I felt that it was my duty towards my child to get them vaccinated.* (Malgorzata)

The mandatory nature of the Polish vaccination program seemed to be the most important difference when the participants compared the two programs. However, those who had children born in Poland prior to moving to Norway and seemed more familiar with the differences between the programs, also pointed out that despite being based on global recommendations, there were differences in cost associated with some vaccines:

*“I think that there are not big differences*,* because both Poland and Norway follow the global [vaccine] recommendations. The healthcare system is public*,* so I think the recommendations are similar. In practice though*,* it may look a little different because in Poland there were some vaccines for children that cost money.”* (Zofia)

## Discussion

This study explored attitudes towards childhood vaccination in Norway among Polish immigrants. Our study focused on the participants’ personal experiences and views of childhood vaccination in general, as well as differences in experiences in their birth country versus their country of residence. We found that all the participants in this study had vaccinated their children, either in Poland, Norway, or a combination of the two programs. They explained their decisions with a need to ensure their children are protected against diseases, as well as concerns about their children’s health. Although some of the participants expressed hesitancy about certain vaccines, the overall sentiment in the interviews were positive views of childhood vaccination and trust in vaccines, health care providers and health care authorities. The influence of parents’ trust in medicine, advice from health care providers and authorities, as well as knowledge about vaccine have previously been identified as being important for parents decision-making process and views of childhood vaccination [29].

Nevertheless, some of the participants expressed hesitancy towards certain vaccines their children had been offered. Some of this hesitancy might potentially be explained by the differences in recommended childhood vaccines in Poland and Norway. For some, the hesitancy was directed towards vaccines they had never received themselves or vaccines offered in Norway that they believed were not part of the Polish childhood vaccination program. The pneumococcal vaccine was added to the Polish childhood vaccination program in 2017 [30], which was after many of the participants had relocated to Norway. This timing might have resulted in participants perceiving the vaccine as new or being unaware of the current vaccination guidelines in Poland. Furthermore, hesitancy towards the HPV vaccine was also expressed by some participants emphasizing not knowing anyone from Poland who had taken it, despite having Norwegian acquaintances that encouraged them to get the vaccine. The difference in views shared by their Polish and Norwegian acquaintances mirrors differences in HPV vaccine uptake based on country background seen in previous studies [3, 31, 32], with HPV-vaccine uptake being higher among Norwegians compared to Poles. At the time the study was conducted, HPV vaccines were expensive, not mandatory, and often directed towards females only in Poland [33]. Furthermore, past studies have documented a lack of educational campaigns surrounding HPV in Poland [34] and despite the implementation of a nationwide HPV vaccination program in 2023, a recent study found that one-third of Polish adults were unfamiliar with the program [35]. These factors might be the reason why some participants in the study claimed they did not know anyone who had received the vaccine in Poland [31]. This is also seen in other countries, where girls with Polish background in Scotland have been found to have significantly lower HPV vaccine coverage when compared to girls from the UK [32]. Regarding meningococcal vaccination, previous studies have pointed out poor knowledge about this vaccine among parents in Poland [36], which might influence the lack of familiarity with this vaccine among the participants.

Unlike past studies finding conspiracy beliefs about vaccination to be more common among Polish immigrants in Norway [20], none of the participants in our study expressed beliefs in conspiracy theories related to vaccination. However, two of the participants spoke about so-called “clean” vaccines, which might indicate that discussions surrounding vaccines in Poland are more influenced by antivaccine messages compared to Norway. The reference to “clean” vaccines can be found in previous studies documenting how Twitter bots and internet trolls influence the vaccine discourse with statements such as “only the elite gets clean vaccines” [37]. Speculations about differences in the composition of vaccines have also been found among Polish immigrants in Scotland [38], where mandatory Polish vaccines were believed to be polluted or of lesser quality compared to vaccines in the UK.

Comparisons of childhood vaccination in Poland and Norway was evident in many of the interviews, and suggest, like past studies [38], that Polish immigrants compare the childhood vaccines offered in their country of residence with those offered in Poland. As all participants in this study moved to Norway as adults, their opinions are shaped not only by their experiences in Norway, but also in Poland. Thus, their familiarity with two different countries enables them to compare vaccination practices to a greater extent than those who have lived in Norway all their lives. Their decision-making process when considering vaccines for their children was influenced by both Polish and Norwegian sources, including information from health care professionals, family, and friends [38]. This was evident in their decisions about specific vaccines, as well as in their preferences related to type of childhood vaccination programs, with most of the participants in this study preferring the voluntary and free nature of the NCIP to the mandatory PCIP, similar to findings from a Scottish study of Polish immigrants favoring voluntary vaccination [38].

Past studies have found that children born in Norway to Polish parents have lower vaccination coverage for vaccines in the NCIP compared to the general population and for Polish immigrants in Norway to have lower trust in the Norwegian healthcare system and hold less positive attitudes towards vaccines such as COVID-19 [18, 19]. However, the participants in our study were mostly positive about childhood vaccines and expressed having trust in both the Norwegian healthcare authorities and system. Furthermore, previous studies from Norway have found that higher income and education might be associated with higher vaccination coverage [15] and as the majority of the participants in this study had lived in Norway for many years and had higher education, this could contribute to the participation in the NCIP. In addition, most of our participants were proficient in the Norwegian language, which might make information about childhood vaccines more accessible and remove language barriers identified in previous studies [21]. The language proficiency, years of residency and education level of the sample of participants suggests that the study reached a part of the Polish immigrant population in Norway who might be more familiar with the NCIP and the Norwegian health care system, compared to more recently arrived immigrants.

### Limitations

The 15 participants in our study were all well-integrated in Norway, with the majority having completed higher education (*n* = 12) and had lived in Norway for more than 10 years (*n* = 11). With one exception, all participants had moved to Norway on a permanent basis. Their demographics are not representative of all Polish migrants to Norway. Having lived in Norway for a longer time likely makes them more familiar with the Norwegian health care system, and the overall high education level could also indicate that they are more health conscious than Polish migrants in general. Most participants described vaccination as something they were familiar with, and no one expressed having difficulties navigating the Norwegian health care system. Future studies would benefit from exploring the experiences of Polish migrants with more variation in education level and years of residency in Norway. Furthermore, the study was conducted in Oslo and surrounding suburbs, thus further studies exploring the experiences of Polish migrants in other parts of Norway would be of value. Lastly, as we wanted to ensure that language was not a barrier to participating in the study, all participants could choose the language they preferred to be interviewed (Polish, Norwegian or English). As all fifteen preferred Polish and only one of the researchers spoke Polish, all interviews were done by the same researcher.

### Recommendations

The study highlights the importance of ensuring that parents have enough information to make a fully informed choice about childhood vaccination. This might be especially important for parents who are familiar with the vaccination schedules in several countries. In fact, lack of knowledge or unfamiliarity with certain vaccines in the NCIP had resulted in some participants declining the vaccines.

## Conclusion

The participants in this study expressed positive views of childhood vaccination in general and viewed it as a choice they make to protect their children and their health. Few expressed hesitancy towards vaccines in the NCIP and among those who had declined certain vaccines, this was not associated with expression of anti-vaccine sentiments or beliefs in conspiracy theories, but more often related to lack of knowledge or unfamiliarity with a specific vaccine in the NCIP. This highlights the importance of ensuring that parents receive enough information about each vaccine that is offered to their child, especially if parents are familiar with childhood vaccination schedules differing from the NCIP. Comparisons of vaccination in Poland and Norway were common and it was evident that their opinions about vaccination were shaped by experiences from both their birth country and their country of residence. Despite their positive views of childhood vaccination and decisions to have their children vaccinated, most of the participants preferred the voluntary nature of the NCIP as compared to the mandatory PCIP.

## Data Availability

No datasets were generated or analysed during the current study.

## Abbreviations

NCIP: Norwegian Childhood Immunization Program
HPV: Human Papilloma Virus
WHO: World Health Organization
EU: European Union
PCIP: Polish Childhood Immunization Program
NIPH: Norwegian Institute of Public Health
DPIA: Data Protection Impact Assessment
